# Healthcare Professionals’ Knowledge and Behaviors Regarding Drug–Dietary Supplement and Drug–Herbal Product Interactions

**DOI:** 10.3390/ijerph19074290

**Published:** 2022-04-03

**Authors:** Zorica Stanojević-Ristić, Isidora Mrkić, Aleksandar Ćorac, Mirjana Dejanović, Radoslav Mitić, Leonida Vitković, Julijana Rašić, Dragana Valjarević, Aleksandar Valjarević

**Affiliations:** 1 Department of Pharmacology and Toxicology, Faculty of Medicine, University of Priština-Kosovska Mitrovica, 38220 Kosovska Mitrovica, Serbia; radoslavmitic@yahoo.com (R.M.); julijana.rasic@med.pr.ac.rs (J.R.); 2 Department of Ophthalmology, Medical Center, 38220 Kosovska Mitrovica, Serbia; isidorar@gmail.com; 3 Department of Preventive Medicine, Faculty of Medicine, University of Priština-Kosovska Mitrovica, 38220 Kosovska Mitrovica, Serbia; drcorac@gmail.com; 4 Department of Physiology, Faculty of Medicine, University of Priština-Kosovska Mitrovica, 38220 Kosovska Mitrovica, Serbia; mirjana.dejanovic@gmail.com; 5 Department of Histology and Embryology, Faculty of Medicine, University of Priština-Kosovska Mitrovica, 38220 Kosovska Mitrovica, Serbia; leonidavitkovi@yahoo.com; 6 Department of Mathematics, Faculty of Sciences and Mathematics, University of Priština-Kosovska Mitrovica, 38220 Kosovska Mitrovica, Serbia; dragana.valjarevic@pr.ac.rs; 7 Department of Geospatial and Environmental Science, Faculty of Geography, University of Belgrade, 11000 Belgrade, Serbia; aleksandar.valjarevic@gef.bg.ac.rs

**Keywords:** drug–dietary supplement interactions (DDSIs), drug–herbal product interactions (DHPIs), knowledge, behavior, healthcare professionals (HCPs)

## Abstract

Given the widespread use of dietary supplements (DS) and herbal products (HP), healthcare professionals (HCPs) will increasingly encounter patients who use these preparations with conventional drugs and who need their services to reduce the consequences of adverse therapeutic outcomes. The aim of our survey was to assess the knowledge and behaviors of HCPs regarding the risk of potential drug–dietary supplement (DDSIs) and drug–herbal product (DHPIs) interactions. This cross-sectional survey collected data via on paper-based questionnaire among general practitioners (GPs) (*n* = 105), specialty doctors (*n* = 87) and nurses (*n* = 154). The HCPs were mostly familiar with the interaction of doxycycline with magnesium (83%) and were least familiar with interaction of warfarin with glucosamine (14%). The results on DDSIs and DHPIs knowledge showed that GPs scored significantly higher than nurses (*p* < 0.001 and *p* = 0.003, respectively), while specialty doctors scored significantly higher than nurses only on DDSIs knowledge (*p* < 0.001). Only 28% of respondents reported that they often or always ask patients on drug therapy about the use of DS or HP, and 25% of respondents record such data in the medical documentation of patients. Our results showed that HCPs have sufficient knowledge about most major DDSIs and DHPIs, but insufficient knowledge about most moderate interactions. However, their overall knowledge and behavior regarding the risk of these interactions indicate the need for further continuing education and training.

## 1. Introduction

The use of dietary supplements (DS) and herbal products (HP) derived from natural substances are increasing continuously worldwide, due to the complementary and alternative medicine popularity. In 2020, it was reported that sales of herbal dietary supplements (HDS) alone in the USA reached 11.2 billion dollars with an annual increase in consumer spending of more than 17% [1]. High rates of herbal and DS use were reported during the COVID-19 pandemic [2].

The context of usage of DS and HP varies widely from country to country. In some countries, their use is limited to protect and improve health while others permit use for medicinal purposes [3]. In addition, the problem with the use of herbs is the lack of consistent terminology. In the USA, DS and HP are not classified as drugs by the Food and Drug Administration (FDA). The Dietary Supplement Health Education Act (DSHEA) [4] from 1994 defined DS as a specific category of food intended to supplement the diet that is not represented as conventional food. Herbs or other botanicals are specifically mentioned as DS. In the EU, herbal medicines (HM) are used based on a tradition of use. The EU Directive 2004/24/EG [5] defined guidelines for registering HM for self-medication. These medicinal products must have a sufficiently established tradition and be proven to be safe enough to be placed on the over the counter (OTC) market [6,7]. Besides HM, the EU allows the market of vitamins, minerals, botanicals, and other substances as food supplements. In the Republic of Serbia, HP is sold as herbal medicines, traditional herbal, or homeopathic medicines [8], or as HDS [9].

It is recognized that DS and HP can cause adverse effects [10,11,12]. Common causes of such events are adulteration of DS and HP with active pharmaceutical ingredients which was approved or removed from the market due to their toxicity. Sexual enhancement, weight loss, and sports supplements are among the most common products adulterated with these substances [13,14,15]. Some plants used to improve physical performance, such as ginseng, caffeine, and green tea can be contaminated with agents banned in sports [16]. Contamination of sports supplements with undeclared doping substances poses a major health risk to consumers [17]. Serious adverse reactions to these products, such as intracerebral hemorrhage, cardiac failure, and death, have been reported [18]. 

The safety of DS and HP has become an important issue for health regulatory authorities. In this regard, an area that has become increasingly important is that of drug-DS (DDSIs) and drug-HP interactions (DHPIs). Published data showed that between 21% and 43% of patients who take prescription medications utilize DS concurrently [19,20]. Patients with comorbidities, additional medications, and genetic polymorphisms, are at particular risk for DDSIs and DHPIs [21,22]. 

Pharmacokinetic and pharmacodynamic processes have been found to play an important role in DDSIs and DHPIs, but their mechanisms are not fully understood [23]. Pharmacokinetic interactions occur when DS or HP shares the same mechanism of absorption, distribution, metabolism, or excretion as a co-administered drug. Most of these interactions typically manifest through the induction or inhibition of drug-metabolizing enzymes (cytochrome P450, CYP), or either chelation or intestine alteration of drug transporters (P-glycoprotein) which influences the absorption and bioavailability of the medication [24,25,26]. Pharmacodynamic interactions have been less studied and may be additive (or synergetic), or antagonistic, whereby DS or HP potentiate or reduce the pharmacological action of drugs [27]. Understanding this issue by health care providers is essential to minimize the risk of potentially harmful combinations and to maximize benefits when treating a disease. 

The timing of drug administration can be an important factor that influences the occurrence of DDSIs and DHPIs. The co-administration with DS or HM may decrease drugs absorption, and their oral bioavailability, thus affecting the efficacy of such medicines. The mechanisms that decrease drugs absorption include complex formation, increased pH, or decreased enterohepatic circulation [28]. For example, divalent or trivalent cation-containing DS such as iron, calcium, zinc, and magnesium, may interact with many drugs and reduce their efficacy. The clinical implications of these interactions are therapeutic failures. Therefore, the knowledge of the timing of drug administration in relation to DS could reduce the incidence of these interactions.

Additionally, the growing interest in DS and HP has not been followed by communication improvement between users and health care providers on this topic. Among herbal and DS users with chronic conditions, less than 51% disclosed use to their conventional health care provider [29]. Several studies have shown that patients’ nondisclosure of supplement use when they feel their conventional medical providers are disinterested, disapprove, respond negatively to their use, or do not ask [30,31,32]. In order to provide a more adequate health-care service to such patients, practitioners should be familiar with commonly occurring DDSIs and DHPIs. 

Currently, little is known about the knowledge of health care professionals (HCPs) regarding DDSIs and DHPIs. 

The main objective of this study was to evaluate the knowledge of HCPs about the potential and common DDSIs and DHPIs and to examine their behavior regarding the risk of these interactions.

## 2. Materials and Methods

### 2.1. Study Design and Participants

A descriptive, cross-sectional survey was conducted between May and July 2021 at the Medical Center in Kosovska Mitrovica and the Faculty of Medicine, the University of Pristina in Kosovska Mitrovica, Serbia. The data were collected using an anonymous self-administered questionnaire. The study protocol was approved by the Ethics Board and Ethics Committee of these institutions. 

The respondents were the HCPs at the Medical Center (general hospital and health centers) and the Faculty of Medicine in Kosovska Mitrovica. The HCPs on postgraduate training in the Faculty of Medicine, such as nursing students, doctoral students, and medical residents were also included in the study. The total number of HCPs was 482, of whom 136 were excluded because of incomplete data. Stratified sampling was performed according to the profession, in which HCPs were divided into subgroups: general practitioners (GPs), specialty doctors (including subspecialty doctors), and nurses. 

### 2.2. Questionnaire

A survey questionnaire was designed based on a literature review and similar research previously conducted [33,34,35,36]. The used DS and medicines were the commonly available products and prescribed drugs in the Serbian market. 

The information on the potential and known DDSIs and DHPIs were obtained using the MEDLINE database (via PubMed) [37], a published textbook [38], and available web-based drug interaction checker tools (Drugs.com and Medscape). Pairs of identified DDSIs and DHPIs were classified into levels, according to clinical relevance/severity of drug interactions (i.e., major, moderate, minor). Major DDSIs and DHPIs were categorized as “contraindicated”, and as “serious-avoid co-administration”. Moderate DDSIs and DHPIs were “use with caution-monitor”. DDSIs and DHPIs with a severity rating of “major” and “moderate” were selected for this study. 

The respondents were invited to participate in the survey during their rest break at work and after attending a lecture at the Faculty of Medicine (HCPs on postgraduate training). We used a paper-based questionnaire designed where the respondents could select one answer or enter the appropriate data. One of the research team members first explained the purpose of the survey to respondents and then assured them that all information would be completely confidential. Respondents who agreed to participate voluntarily were given consent forms and questionnaires to complete without using mobile applications, the internet, or any assistance. During that time, research team members were available if assistance was required. 

The questionnaire consisted of four sections. The first section contained 7 questions regarding respondents’ demographic and professional characteristics, such as age, gender, type of practice, specialty, length of practice experience, postgraduate training, and previous education/training about DS or HP. 

The second section included a list of 10 questions designed to assess respondents’ knowledge about DDSIs. The major DDSIs categorized as “serious” were interactions of fosinopril with potassium, levofloxacin with iron, and doxycycline with magnesium. All others were moderate DDSIs: levothyroxine with calcium, zolpidem with melatonin, warfarin with glucosamine, levodopa with pyridoxine, hydrochlorothiazide with vitamin D3, warfarin with coenzyme Q10, and acetylsalicylic acid (ASA) with omega-3 fatty acid. The third section contains 10 different questions about DHPIs. The major DHPIs categorized as “contraindicated” was the interaction of indinavir with St John’s wort (*Hypericum perforatum*), while as “serious” were interactions of cyclosporine with St John’s wort and phenobarbital with valerian (*Valeriana officinalis*). Moderate DHPIs were ASA with ginger (*Zingiber officinale*), atorvastatin with black cohosh (*Actea racemosa*), warfarin with ginseng (*Panax ginseng*), insulin with aloe vera (*Aloe* spp.), ASA with ginkgo (*Ginkgo biloba*), warfarin with cranberry (*Vaccinium* spp.), and itraconazole with echinacea (*Echinacea* spp.). Respondents were asked to answer on DDSIs and DHPIs questions as “yes”, “no”, or “don’t know”. One mark was given to each question answered correctly. The summation of the marks was labeled as the knowledge score for each section. 

The fourth section included 6 questions about respondents’ behaviors regarding the risk of DDSIs and DHPIs. Behaviors were assessed by questions related to the frequency of questioning patients on drug therapy about their use of DS or HP, as well as OTC medications, how often they document such information, and how often recommend patients on drug therapy to take these products. These issues also included questions about respondents’ assessment of knowledge about DS and HP and whether that knowledge is sufficient to manage interactions, as well as the importance of DDSIs and DHPIs education for their future practice. Respondents selected one answer from a 5-point Likert scale which ranged from none/never (1), rarely (2), sometimes (3), often (4) to always (5). 

### 2.3. Statistical Analysis

The statistical analysis of the obtained results was made using SPSS statistical software package version 21.0 (IBM Corp., Armonk, NY, USA). Frequencies and percentages were used to describe categorical variables. Due to the scale of measurement, the scores were compared using Kruskal-Wallis and Mann-Whitney U test. These non-parametric tests were applied because the data did not have a normal distribution. Statistical analysis was performed to determine the association between the overall knowledge score of DDSIs/DHPIs and respondents’ general characteristics. Results were considered to be statistically significant when the *p*-value was less than 0.05.

## 3. Results

### 3.1. Characteristics of Study Respondents

Among the 346 HCPs who completed the survey, 30% (*n* = 105) were general practitioners (GPs), 25% (*n* = 87) were specialty doctors and 45% (*n* = 154) were nurses. The distribution of type of practice and specialties are listed in Table 1. Of the specialty doctors, 66% were in the age range of 40 to 49, as were 38% of the nurses, while 64% of GPs were younger than 39 years. The most of GPs and nurses were female (63% and 66%, respectively), while over half of the specialty doctors were male (51%). Fifty-four percent of HCPs had been qualified as nurses for more than 21 years, 57% as specialty doctors for 11 to 20 years, and 58% as GPs for less than 10 years. Of the total number of HCPs, 55% were on postgraduate training (which includes nursing students, doctoral students, and medical residents). The research showed that 22% of specialty doctors, 12% of GPs, and 5% of nurses had professional education or training (including continuing medical education, conferences, lectures, courses) related to DS or HP.

### 3.2. Respondents Knowledge about DDSIs

The majority of HCPs showed a high level of knowledge of major DDSIs, such as the interaction of doxycycline with magnesium (83%), and levofloxacine with iron (71%). Similarly, more than half (57%) of HCPs were aware of the interaction of fosinopril with potassium.

However, less than half of HCPs were aware of the moderate DDSIs between ASA with omega-3 fatty acid (43%), hydrochlorothiazide with vitamin D3 (40%), levothyroxine with calcium (37%), warfarin with coenzyme Q10 (37%), zolpidem with melatonin (34%). Moreover, only a small percentage of HCPs answered correctly about the moderate DDSIs of levodopa with pyridoxine (18%) and warfarin with glucosamine (14%). 

Nurses showed a slightly higher percentage of correct answers compared to GPs and specialty doctors regarding the interactions of fosinopril with potassium, and levodopa with pyridoxine. The frequency of correct DDSI responses among HCPs is summarized in Table 2.

### 3.3. Respondents’ Knowledge about DHPIs

The respondents were most familiar with major DHPIs of phenobarbital with valerian (61%). However, only a small number of HCPs were able to identify the interactions involving St John’s wort with indinavir (19%) and cyclosporine (18%), categorized as “contraindicated” and “serious”. 

On the other hand, more than half of the respondents correctly identified the moderate DHPIs of ASA with ginger (71%), ASA with ginkgo (64%), and warfarin with ginseng (56%). Approximately one-third of respondents answered correctly about the interactions of itraconazole with echinacea (32%), atorvastatin with black cohosh (29%), warfarin with cranberry (28%), and insulin with aloe vera (21%). The HCPs responses of the DHPIs are summarized in Table 3.

### 3.4. Respondents’ Knowledge Score about DDSIs and DHPIs

The result of DDSIs knowledge has shown that GPs and specialty doctors scored significantly higher than nurses (*p* < 0.001). Moreover, GPs scored significantly higher than nurses (*p* = 0.003) in the section on DHPIs knowledge. Specialty doctors scored higher on DDSIs than GPs, but lower on DHPIs. These differences were not significant (see Table 4).

### 3.5. Relationship between Respondents’ Characteristics with Their Overall Knowledge Score on DDSIs and DHPIs

Characteristics such as age, length of practice experience, and previous education or training about DS or HP had a significant effect (*p* < 0.001) on the level of knowledge of HCPs about DDSIs, and DHPIs (see Table 5). Overall knowledge of DDSIs and DHPIs was not associated with gender, type of practice, and postgraduate training.

### 3.6. Respondents’ Behaviors Regarding the Risk of DDSIs and DHPIs

The behaviors of HCPs regarding the risk of DDSIs and DHPIs are given in Table 6. Overall, 28% of respondents reported that they often or always ask patients on drug therapy about their use of DS or HP, and 49% of respondents ask about the use of OTC medication. Recording of these data in the medical documentation of patients were slightly higher in GPs (37%) compared to specialty doctors (23%) and nurses (17%). Nurses were more likely (64%) to often recommended the use of DS or HP to patients on drug therapy than GPs (45%) and specialty doctors (33%). However, only 31% of specialty doctors, 24% of GPs, and 12% of nurses believe that they often have enough knowledge about DS and HP to manage DDSIs and DHPIs. Finally, although most HCPs stated that they did not receive any professional education or training on DS and HP, 76% of GPs, 61% of specialty doctors, and 55% of nurses believe that education on this topic will often be useful and necessary to them in future practice. There were no significant differences between study groups.

## 4. Discussion

This study evaluated the knowledge and behaviors regarding DDSIs and DHPIs among three groups of HCPs (GPs, specialty doctors, and nurses). Based on the HCPs’ response, it can be seen that GPs, specialists, and nurses were more familiar with major than moderate DDSIs and DHPIs. In general, HCPs were not familiar with some clinically relevant DDSIs and DHPIs included in this survey. For the two major DHPIs, over 80% of HCPs answered incorrectly. Moreover, for eleven moderate DDSIs and DHPIs, more than half of HCPs gave an incorrect answer. Similar results were obtained in the study by Al-Arifi et al. (2016), which noted the lack of knowledge of warfarin-herbal medicines interactions among HCPs [33].

For the major DDSIs, HCPs have shown an adequate level of knowledge. The majority of GPs, specialty doctors, and nurses identified the interactions of antibiotics doxycycline and levofloxacin with magnesium and iron. Co-administration of these antibiotics with magnesium and iron at the same time and inappropriate separation may lead to clinically significant decreases in oral antibiotics bioavailability [39]. Tetracyclines and fluoroquinolones interact with polyvalent cations-containing DS and form complex with poor absorption. Spacing out the administration times is a common recommendation to avoid these interactions [39]. Contrary to our findings, Eljaaly et al. (2021) revealed that interactions between fluoroquinolones or tetracyclines with divalent or trivalent cation-containing compounds can still occur frequently in clinical settings [40]. 

Another major DDSIs, the interaction of fosinopril with potassium was recognized in more than half of HCPs. Fosinopril can elevate potassium plasma levels by interfering with the production and/or secretion of aldosterone [41]. Concomitant intake of angiotensin-converting enzyme (ACE) inhibitors with potassium-containing supplements leads to potentially serious hyperkalemia. The medical consequences of this interaction are well known.

Among the major DHPIs, more than half of HCPs were familiar with the interaction involving phenobarbital and valerian. For phenobarbital, co-administration with valerian may result in enhanced CNS depression by added drug effects [38]. This effect prolongs phenobarbital-induced sleep time and can lead to serious undesirable outcomes. 

On the other hand, the lack of knowledge about the other major DHPIs between antiretroviral indinavir and immunosuppressant cyclosporine with St. John’s wort is evident. Intake of St John’s wort induces CYP3A4 isozyme in the liver and intestine [22]. Because HIV protease inhibitors and immunosuppressants are substrates of this isozyme, induction by St John’s wort leads to the development of drug resistance and treatment failure [42,43]. Patients with HIV are high users of HP [44] but rarely disclose their use to health care providers [45]. Self-administration of St. John’s wort in transplant patients was the cause of organ rejection [46]. This indicates that insufficient knowledge of DDSIs and DHPIs by HCPs usually may not provide relevant information to patients and result in adverse medical consequences. Moreover, inadequate patient awareness of this topic may result in subsequent disease progression and serious medical outcomes.

The HCPs’ knowledge about moderate DDSIs was inadequate. More than half of the respondents were not aware of the interaction of ASA with omega-3 fatty acids, hydrochlorothiazide with vitamin D3, levothyroxine with calcium, warfarin with coenzyme Q10, and zolpidem with melatonin. Concomitant use of ASA and omega-3 fatty acids enhance the antiplatelet effects of aspirin [47]. In this regard, antiplatelet drugs interact with fish oil and other products that contain omega-3 fatty acids increasing the patient’s risk of bleeding [48]. Moreover, both hydrochlorothiazide and vitamin D3 contribute to the retention of calcium in the body and the development of hypercalcemia, but with different mechanisms. Thiazide diuretics increase calcium reabsorption in kidneys, while vitamin D3 increases the absorption of calcium in the gut [49]. Concomitant intake of levothyroxine with calcium supplements reduces the absorption of levothyroxine, leading to treatment failure in patients with hypothyroidism [50,51]. Separating the administration of levothyroxine and calcium-containing DS should ensure that the efficacy of levothyroxine is maintained. 

Coenzyme Q10 (known as ubiquinone) is chemically similar to K-vitamins, which competes with and counteracts warfarin’s anticoagulation effects. For warfarin, co-administration with coenzyme Q10 may result in a decreased response to warfarin and predispose patients to clots [52]. Moreover, using zolpidem concurrently with melatonin potentiates their hypnotic effects [38]. This interaction can increase sedation and dramatically impair patient coordination. Therefore, the lack of knowledge of HCPs about these interactions cannot prevent such undesirable outcomes. 

A majority of respondents were unfamiliar with the other moderate interactions involving levodopa with pyridoxine and warfarin with glucosamine. Glucosamine is among the most commonly used DS by the elderly [53], a group often prescribed warfarin for the prevention of thromboembolic complications. The intake of warfarin and glucosamine concomitantly increases the patients’ risk of bleeding [54]. Since glucosamine is a component of heparin, it is possible that have an additive pharmacodynamic effect on coagulation with anticoagulants. The availability of levodopa to the brain parenchyma decreases with co-administration of pyridoxine due to an increase in the excretion of major levodopa metabolites [55]. The combination of levodopa and carbidopa minimized the effects of pyridoxine. Interestingly, nurses were more familiar with the interaction of fosinopril with potassium and levodopa with pyridoxine compared to specialty doctors and GPs. This better knowledge of the interactions could be due to the more frequent administration of these drugs in the practice of this professional group. Because nurses have a unique role as caregivers, educators, and administrators of drugs, they are particularly well-positioned to prevent and alert for potentially life-threatening drug-related problems [56].

St. John’s wort, ginkgo, echinacea, garlic, ginseng, and ginger are widely popular and known substances that cause drug interactions [57,58]. The most commonly interacting drugs were antiplatelet agents and anticoagulants [59]. Herbs, such as ginkgo and ginger, are already known to potentiate the antithrombotic effect of aspirin, inhibiting platelet function [60]. For warfarin, co-administration with ginseng reduces its anticoagulant effect by induction of hepatic P450 enzyme system activity [61]. In this survey, HCPs showed adequate knowledge regarding the moderate interactions of ASA with ginger and ginkgo, as well as the interaction of warfarin with ginseng. Their awareness about such interactions could prevent serious adverse reactions associated with platelet, bleeding, and clotting disorders.

However, the HCPs knowledge about moderate DHPIs was insufficient. The majority of respondents were unfamiliar with interactions of itraconazole with Echinacea, atorvastatin with black cohosh, warfarin with cranberry, and insulin with aloe vera. Echinacea simultaneously with itraconazole may reduce the systemic bioavailability of itraconazole by induction of CYP3A4 izoenzyme. The mechanism of this interaction is still unclear, because of inconsistent data [62,63]. The intake of warfarin with cranberry concomitantly may increase the International Normalized Ratio (INR) and risk of bleeding. [64,65]. Due to the additive hepatotoxic effect, the use of atorvastatin concomitantly with black cohosh may lead to an elevation of liver enzymes [66]. Moreover, insulin and aloe vera have an additive blood-glucose-lowering effect, so their co-administration may provoke symptoms of hypoglycemia [67]. Therefore, poor knowledge of these interactions can lead to compromised patient safety.

The assessment of knowledge scores among HCPs showed that GPs and specialty doctors had higher DDSIs and DHPIs scores compared to nurses. This is consistent with earlier reports where nurses tended to score the lowest among the groups [33,68]. Scores of specialty doctors were slightly higher than those of GPs in DDSIs but were lower in DHPIs knowledge. A possible explanation for this is that GPs are more familiar with potential DDSIs and DHPIs that include medications they prescribe more frequently. Specialty doctors usually have a better knowledge of drugs in their specialty areas. In our survey, younger HCPs and those with less working experience were more able to identify potential DDSIs and DHPIs. This corroborates with earlier published survey by Glassman et al. (2002) where younger clinicians had better knowledge about drug interactions than older clinicians [69]. Moreover, previous education or training had a significant effect on the extent of knowledge about DDSIs and DHPIs. Similarly, Charrois et al. (2007) reported that pharmacists with training in natural health products (NHP) had a significantly higher comfort level in counseling about NHP-drug interactions [36]. As in our survey, nurses were the professional group with the greatest need for education and training on this topic [70]. 

Additionally, nurses were more likely to routinely recommend the use of DS or HP to patients on drug therapy. This is a serious concern because the concomitant use of DS or HP with OTC or prescription drugs increases the risk of potential DDSIs and DHPIs, especially if knowledge of this topic is insufficient. 

In our sample, less than one-third of HCPs regularly asked patients on drug therapy about their use of DS or HP and recorded such data in patients’ medical documentation. However, HCPs were more likely to ask patients about the use of OTC drugs than DS or HP. Clearly, HCPs may be aware that patients are using DS or HP, but they are still not treating discussion about these products in the same manner as other types of medications [71]. 

The survey results also showed that respondents generally felt that knowledge of DS and HP was often not enough for them to manage DDSIs and DHPIs. It is encouraging that most HCPs are aware that continuous education on this topic will be their necessity in future practice. 

There are limitations to this survey. Data were collected from the Medical Center and the Faculty of Medicine in Kosovska Mitrovica and are not representative of the entire population of HCPs in Serbia. The study did not involve hospital and clinical pharmacists. Moreover, the questionnaire did not include questions about available web-based drug interaction checker tools which HCPs use in clinical practice. Further research is needed on a larger sample, including more medical centers, pharmacists, a larger number of potential and known DDSIs and DHPIs, and HCPs with educational intervention.

## 5. Conclusions

The growing trend of DS and HP use by an increasing number of patients, who take these products simultaneously with conventional medications, arouses an interest in the problem of DDSIs and DHPIs. The potential dangers associated with these interactions, such as adverse drug reactions, toxicity, or loss of treatment efficacy, are known and can be managed rationally. 

This research indicates that HCPs have better knowledge on major DDSIs and DHPIs. However, overall, the HCPs’ relative lack of knowledge suggested that they are unfamiliar with most DDSIs and DHPIs. HCPs are aware of their inadequate knowledge on this topic, as well as the need for continuous education on DS and HP for their future practice. However, most HCPs do not routinely ask patients on drug therapy about their use of DS and HP and less frequently routinely record such information in patients’ medical records. Therefore, continuing medical education and training of HCPs about harmful DDSIs and DHPIs and their potential to cause negative patient-related outcomes is a necessity to improve HCPs’ knowledge and awareness of this topic. 

## Figures and Tables

**Table 1:** Demographic and professional characteristics of respondents.

Characteristics	GPs	Specialty Doctors	Nurses	Total
	(*n* = 105)	(*n* = 87)	(*n* = 154)	(*n* = 346)
	*n* (%)	*n* (%)	*n* (%)	*n* (%)
Gender				
Male	39 (37)	44 (51)	52 (34)	135 (39)
Female	66 (63)	43 (49)	102 (66)	211 (61)
Age				
≤29 years	33 (31)	0 (0)	34 (22)	67 (19)
30–39 years	35 (33)	9 (10)	31 (20)	75 (22)
40–49 years	19 (18)	57 (66)	59 (38)	135 (39)
≥50 years	18 (17)	21 (24)	30 (20)	69 (20)
Type of practice				
Health center	65 (62)	38 (44)	73 (47)	176 (51)
Hospital	40 (38)	49 (56)	81 (53)	170 (49)
Specialty				
General/family medicine	65 (62)	15 (17)	66 (43)	146 (42)
Internal medicine	19 (18)	40 (46)	30 (19)	89 (26)
Others ^a^	21 (20)	32 (37)	58 (38)	111 (32)
Years of practice experience				
1–10	61 (58)	24 (28)	47 (30)	132 (38)
11–20	25 (24)	50 (57)	24 (16)	99 (29)
≥21	19 (18)	13 (15)	83 (54)	115 (33)
Postgraduate training (yes)	67 (64)	34 (39)	89 (58)	190 (55)
Professional education or training about DS and HP (yes)	13 (12)	19 (22)	8 (5)	40 (12)

GP_S_—general practitioners. ^a^ Include: ophthalmology, obstetrics and gynecology, surgery, dermatology, psychiatry, and neurology.

**Table 2:** Frequency of correct answers about drug-dietary supplement interactions (DDSIs) among respondents.

Question ^a^	GPs Correct Answer *n* (%)	Specialists Correct Answer *n* (%)	Nurses Correct Answer *n* (%)	Total Correct Answer *n* (%)
Major DDSIs—Serious-avoid co-administration				
Doxycycline with magnesium	92 (88)	81 (93)	114 (74)	287 (83)
Levofloxacin with iron	89 (85)	62 (71)	94 (61)	245 (71)
Fosinopril with potassium	42 (40)	43 (49)	111 (72)	196 (57)
Moderate DDSIs—Use with caution-monitor				
ASA with omega-3 fatty acid	46 (44)	57 (65)	47 (31)	150 (43)
Hydrochlorothiazide with vitamin D3	46 (44)	48 (55)	43 (28)	137 (40)
Levothyroxine with calcium	67 (64)	39 (45)	23 (15)	129 (37)
Warfarin with coenzyme Q10	25 (24)	36 (41)	68 (44)	129 (37)
Zolpidem with melatonin	41 (39)	40 (46)	37 (24)	118 (34)
Levodopa with pyridoxine	8 (8)	7 (8)	47 (31)	62 (18)
Warfarin with glucosamine	25 (24)	12 (14)	10 (6)	47 (14)

^a^ See File S1 in Supplementary Materials. GPs—general practitioners.

**Table 3:** Frequency of correct answers about drug-herbal product interactions (DHPIs) among respondents.

Question ^a^	GPs Correct Answer *n* (%)	Specialists Correct Answer *n* (%)	Nurses Correct Answer *n* (%)	Total Correct Answer *n* (%)
Major DHPIs—Contraindicated				
Indinavir with St John’s wort	16 (15)	33 (38)	16 (10)	65 (19)
Major DHPIs—Serious-avoid co-administration				
Phenobarbital with valerian	75 (71)	47 (54)	89 (58)	211 (61)
Cyclosporine and St John’s wort	19 (18)	10 (11)	36 (23)	63 (18)
Moderate DHPIs—Use with caution-monitor				
ASA with ginger	82 (78)	52 (60)	110 (71)	244 (71)
ASA with ginkgo	82 (78)	57 (66)	84 (54)	223 (64)
Warfarin with ginseng	59 (56)	53 (61)	80 (52)	192 (56)
Iraconazole with echinacea	35 (33)	34 (39)	41 (27)	110 (32)
Atorvastatin with black cohosh	44 (42)	20 (23)	36 (23)	100 (29)
Warfarin with cranbbery	33 (31)	18 (21)	81 (53)	132 (28)
Insulin with aloe vera	27 (26)	35 (40)	11 (7)	73 (21)

^a^ See File S1 in Supplementary Materials. GPs—general practitioners.

**Table 4:** Respondents’ knowledge score about drug–dietary supplement and drug–herbal product interactions.

	Mean (Median)	*p*-Values ^a^	Pairwise Comparison (*p*-Values ^b^)		
			GPs:Specialty Doctors	GPs:Nurses	Specialty Doctors:Nurses
DDSIs knowledge		<0.001	0.139	<0.001	<0.001
GPs	4.6 (4.0)				
Specialty doctors	4.9 (5.0)				
Nurses	3.9 (4.0)				
DHPIs knowledge		0.014	0.097	0.003	0.503
GPs	4.5 (4.0)				
Specialty doctors	4.1 (4.0)				
Nurses	3.8 (4.0)				

^a^ Kruskal Wallis test for initial multiple group analysis. ^b^ Pairwise comparisons analyzed using Mann Whitney U tests. GPs—general practitioners.

**Table 5:** The association between the overall knowledge score about drug–dietary supplement and drug–herbal product interactions, and respondent’s general characteristics.

Characteristic	Category	Median (Q1–Q3)	Mean (±SD) [Maximum Score 20]	*p*-Value
Gender	Male	8.0 (7.0–10.0)	8.7 (±2.7)	0.095
	Female	8.0 (7.0–10.0)	8.2 (±2.8)	
Age	≤29 years	9.0 (8.0–11.0)	9.2 (±2.7)	<0.001
	30–39	9.0 (7.0–11.0)	8.9 (±2.5)	
	40–49	7.0 (6.0–9.0)	8.0 (±2.7)	
	≥50 years	8.0 (6.0–10.0)	8.1 (±3.1)	
Type of practice	Health center	8.0 (7.0–10.0)	8.5 (±2.8)	0.356
	Hospital	8.0 (7.0–10.0)	8.3 (±2.8)	
Specialty	General/family medicine	8.0 (7.0–10.0)	8.3 (±2.7)	0.08
	Internal medicine	9.0 (7.0–11.0)	8.9 (±3.0)	
	Others ^a^	8.0 (6.0–10.0)	8.2 (±2.7)	
Years of practice experience	1–10	9.0 (7.0–10.0)	9.0 (±2.6)	<0.001
	11–20	8.0 (6.0–11.0)	8.7 (±3.0)	
	≥21	7.0 (6.0–9.0)	7.6 (±2.6)	
Postgraduate training	No	8.0 (7.0–10.0)	8.5 (±3.0)	0.816
	Yes	8.0 (7.0–10.0)	8.4 (±2.6)	
Professional education or training about DS and HP	No	8.0 (7.0–9.25)	8.0 (±2.5)	<0.001
	Yes	11.5 (9.25–13.75)	11.4 (±2.7)	

^a^ Include: ophthalmology, obstetrics and gynecology, surgery, dermatology, psychiatry, and neurology.

**Table 6:** Behaviors of respondents about the risk of drug–dietary supplement and drug–herbal product interactions.

Question ^a^	GPs *n* (%) ^b^	Specialty Doctors *n* (%) ^b^	Nurses *n* (%) ^b^	Total *n* (%) ^b^	*p*-Value ^c^
How often do you ask patients on drug therapy about use of DS or HP?	44 (42)	22 (25)	30 (19)	96 (28)	0.156
How often do you ask patients on drug therapy about use of OTC medication?	68 (65)	52 (60)	51 (33)	171 (49)	0.073
How often have you recommended patients on drug therapy to use DS or HP?	47 (45)	29 (33)	98 (64)	174 (50)	0.168
How often have you noted use of DS or HP in the patient’s medical documentation?	39 (37)	20 (23)	27 (17)	86 (25)	0.583
Is your knowledge of DS and HP sufficient to manage the DDSIs and DHPIs?	25 (24)	27 (31)	18 (12)	70 (20)	0.269
How often is education about DDSIs or DHPIs useful and necessary to you in future practice?	80 (76)	53 (61)	85 (55)	218 (63)	0.067

^a^ Choices for response: 1 = none/ never, 2 = rarely, 3 = sometimes, 4 = often, 5 = always. ^b^ Includes those who responded often and always. ^c^ Kruskal Wallis test for initial multiple group analysis.

## Data Availability

The data presented in this study are available on request from the corresponding author. The data are not publicly available due to ethical reasons that were included in the informed consent form signed by the participants.

## Supplementary Materials

The following supporting information can be downloaded at: https://www.mdpi.com/article/10.3390/ijerph19074290/s1, File S1. Survey questions on the drug-dietary supplement and drug-herbal product interactions.
