# Supplementary material for: Healthcare Professionals’ Knowledge and Behaviors Regarding Drug–Dietary Supplement and Drug–Herbal Product Interactions
Source: Int J Environ Res Public Health. 2022 Apr 3;19(7):4290. doi: 10.3390/ijerph19074290 (PMC8998985; doi:10.3390/ijerph19074290)
Supplement: Supplementary file 1 [file ijerph-19-04290-s001.zip › ijerph-1636903-supplementary.pdf]

**SUPPLEMENTARY FILE S1**

**Survey questions on the drug-dietary supplement and drug-herbal product interactions**

## I Drug-dietary supplement interactions questions

| **Do you think that-** | **Yes** | **No** | **Don't know** |
|---|---|---|---|
| 1. Taking calcium supplements together with levothyroxine may reduce the absorption of levothyroxine, so the use of these supplements is not recommended for at least four hours before or after taking thyroid hormone replacement. | x | | |
| 2. Taking melatonin with zolpidem may increase side effects such as drowsiness, dizziness and confusion, so this combination should be avoided. | x | | |
| 3. Supplements containing iron and/or other minerals can be taken at the same time with levofloxacin. | | x | |
| 4. Taking glucosamine with warfarin enhances the anticoagulant effect of warfarin and increases the risk of bleeding, so this combination should be avoided. | x | | |
| 5. Patients on levodopa should take pyridoxine because it improves the effectiveness of levodopa. | | x | |
| 6. Patients on fosinopril should avoid potassium-containing supplements because of the risk of hyperkalemia. | x | | |
| 7. Hydrochlorothiazide should be avoided in combination with vitamin D3 because they increase the level of calcium in the blood. | x | | |
| 8. Warfarin should be avoided in combination with coenzyme Q10, because it may reduce the effect of warfarin and increase the risk of blood clots. | x | | |
| 9. Patients on Aspirin can take omega-3 fatty acid without any risk of adverse consequences. | | x | |
| 10. Antibiotic doxycycline should not be taken at the same time with magnesium, because this mineral reduces the absorption of doxycycline and its effectiveness. | x | | |

[X] correct answer

**II Drug-herbal product interactions questions**

| Do you think that- | Yes | No | Don't know |
|---|---|---|---|
| 1. Aspirin can be taken together with ginger without any risk of adverse medical consequences. | | x | |
| 2. Taking atorvastatin together with black cohosh can increase the risk of liver damage, so this combination should be avoided. | x | | |
| 3. Patients on warfarin should avoid ginseng because it can reduce the blood level and effect of warfarin. | x | | |
| 4. Patients on insulin can take aloe vera regularly without any risk of subsequent hypoglycemia. | | x | |
| 5. Taking Aspirin together with ginkgo should be avoided due to the increased risk of bleeding. | x | | |
| 6. Patients on warfarin should avoid cranberries because it enhances the anticoagulant effect of warfarin and increases the risk of bleeding. | x | | |
| 7. Phenobarbital can be taken together with valerian without any risk of adverse medical consequences. | | x | |
| 8. Patients on itraconasole should avoid echinacea, becouse this herb may increase the blood levels of itraconazole. | x | | |
| 9. Indinavir can be taken together with St. John's wort, without any risk of loss of treatment efficacy. | | x | |
| 10. Taking St. John's wort together with cyclosporine should be avoided because St. John's wort reduces the blood concentration of cyclosporine and its effectiveness. | x | | |

[x] correct answer
